# Osteogenic stem cell selection for repair and regeneration

**DOI:** 10.1016/j.bonr.2016.01.003

**Published:** 2016-01-28

**Authors:** Marcus Tillotson, Niall Logan, Peter Brett

**Affiliations:** Biomaterials and Tissue Engineering, UCL Eastman Dental Institute, 256 Gray's Inn Road, London WC1X 8LD, United Kingdom.

**Keywords:** Stem cells, Selection, Osteogenic, Titanium, Enrichment

## Abstract

The first osteogenic cells to attach to a titanium (Ti) implant after placement are the multipotent stromal cells (MSCs) that circulate in the bloodstream and are recruited to the site of tissue damage. The reservoirs of these cells are heterogeneous in nature, consisting of a mixture of cells with varying differentiation abilities. In order to utilise these cells and to reduce the chance of unwanted events during regenerative therapies, the selection of a subset of cells that is truly multipotent is required. The behaviour of these cells has been shown to be altered by modifications to Ti implant surfaces, most notably rough, hydrophilic Ti. These changes in behaviour underpin the differences seen in clinical performance of these surfaces. In this study Human bone marrow derived stromal cells (hBMSCs) have been cultured on modified Ti surfaces in order to analyse these changes in cell behaviour. The results demonstrate the different effects of the surfaces and suggest that one surface selectively enriches the population with osteogenic adult ‘stem cells’ by inducing the cell death of the more differentiated cells. Combined with subsequent expansion in bioreactors before implantation, this may lead to a new source of cells for regenerative therapies.

## Highlights

• Different titanium surfaces elicit differing responses from bone marrow derived stromal cells.
• Hydrophilic rough titanium induces increased apoptosis and necrosis in MSCs in vitro.
• Cells selected on rough hydrophilic titanium are more osteogenic than the parent population.
• This may lead to a new source of osteogenic cells for regenerative therapies.

## 1 Introduction

Micro-structured, high surface energy titanium has been shown to be the most effective substrate for osseointegration of an implant with surrounding bone tissue. It has been shown that multipotent stromal cells (MSCs) are present in bone marrow and circulate in the blood stream and that these cells are the first osteogenic cell type recruited to the site of implant placement following trauma or disease (Davies, 1998, Davies, 2003).

Titanium (Ti) and Ti based alloys have been used for dental and orthopaedic implants due to their mechanical and biocompatible properties for many years (Steinemann, 1998, Schuler et al., 2006). This biocompatibility can be attributed to the inertness of the surface oxide layer, unlike other implant materials Ti does not precipitate phosphates and other minerals from bone and has favourable interaction energies with cell surface adhesion proteins. Under atmospheric conditions, a thin oxide layer spontaneously forms on Ti and Ti-alloy surfaces and this has a strong, direct effect on the success of the implant.

The initial stage for osteoblastic cells producing bone tissue is cell adhesion followed by proliferation and differentiation. It has been shown that osteoblastic cell adhesion, growth and differentiation are related directly to surface energy and roughness (Wall et al., 2009, Le Guehennec et al., 2008). Osteoblastic bone cells have been shown to respond with more favourable morphology to roughened surfaces than smooth as well as showing distinct differences in transcriptional regulation of genes that are key to bone-physiology (Brett et al., 2004).

Surface roughness has been an important factor for establishing reliable bone-anchored implants and in vitro studies have provided a positive correlation between surface roughness and cellular attachment as well as subsequent osteoblast-like cell activity (Ronold et al., 2003). This has been supported using in vivo studies that measured the mechanical strength of the connection between bone and implant by torque removal measurements (Buser et al., 1991). Rønold et al. suggested that an upper limit exists for the correlation between surface roughness and bone fixation with an optimum Ra of between 1.0 and 2.0 (Ronold et al., 2003).

Wettability and surface energy are key parameters in the adhesion and spreading of osteoblastic cells. A previous study has suggested that faster healing and greater implant stability was achieved with the rough hydrophilic (SLV) implant surface than around conventional, hydrophobic surfaces. In addition, osteoblastic differentiation was enhanced by the most hydrophilic surface. Wall et al. reported a better osteogenic response to SLV compared to the more hydrophobic SLA surface (Wall et al., 2009). In addition, osteoblastic differentiation was enhanced on the most hydrophilic surface (Khan et al., 2012).

Biomaterials have been shown to provide powerful topographical and chemical cues that can guide cells in the use of regenerative medicine (Dalby et al., 2007, Olivares-Navarrete et al., 2010). This research is based upon the observations that Ti surfaces of differing roughness and wettability exhibit very different effects on bone cells both in vivo and in vitro and that in MSCs cultured in vitro differing levels of apoptosis are induced. It was therefore hypothesised that utilising the nanotopography and chemical signals of novel titanium surfaces to exert selective pressures on stem cell populations might be enriched for cells with osteogenic potential. This has the potential for recruiting highly osteogenic cells for use in repair and regenerative purposes.

This study proposes that the effects of the Ti surfaces on the hBMSCs is due to a change in the make-up of the heterogeneous stem cell population rather than previous in vitro analyses of osteoblastic cells that suggested that contact with Ti causes an enhancement in cellular phenotypic maturation and function (Olivares-Navarrete et al., 2010). These population changes were evaluated by examining phenotypic marker expression and osteogenic mineralisation in hBMSC populations selected on modified Ti surfaces from three unrelated donors over the course of 21 days.

## 2 Materials and methods

### 2.1 Ti surface preparation

Titanium discs with modified surfaces were provided by Institut Straumann AG. The implant surfaces were re-produced on flat discs of 1.8 cm^2^ surface area (15 mm diameter) designed for use in ~ 1.96 cm^2^ 24-well TC-treated plates. There are three surface types; a smoothened, polished surface (SMO), a Sand-blasted Large grit Acid-etched (SLA) and Sand-blasted Large grit Acid-etched hydrophilic surface (SLV). All discs had been prepared from clinically pure Ti. An overview of the methods of fabricating these surface modifications and their physical and chemical characterisations are outlined in Schuler et al. (2006). SMO and SLA discs were passivated prior to use by immersion in 10% *v*/v nitric acid in de-ionised water, air dried and sterilised with UV-irradiation. The smooth surface was modified (SMOds) to have hydrophilic properties by a process of 24 h UV irradiation after passivation. The SLV discs were supplied in sterile saline in glass vials. Tissue culture plastic (TCP) was used as a control to evaluate material related differences.

### 2.2 Selected population procedure

**Fig. 1:**
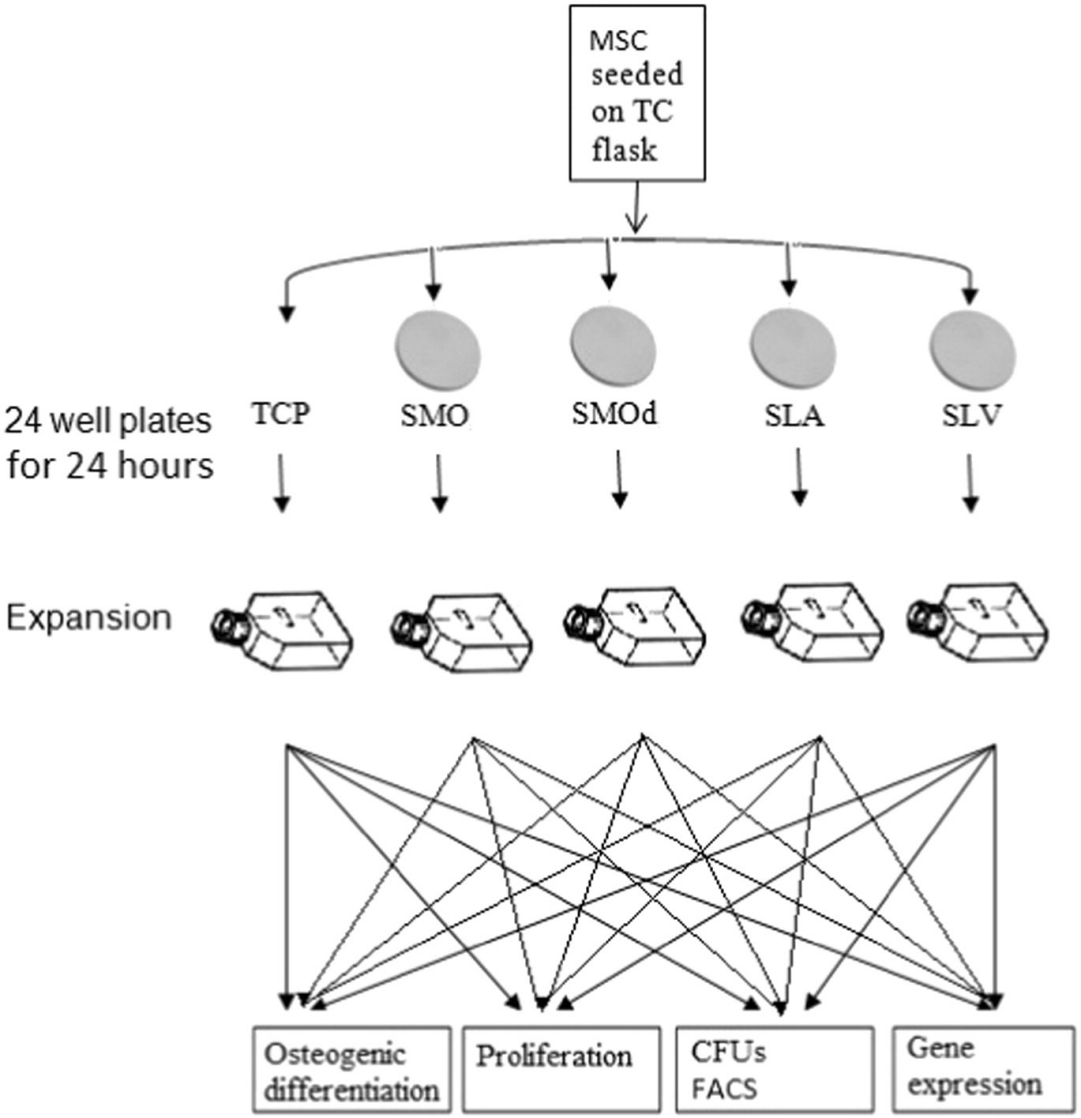
Stem cell selection by modified Ti experimental matrix. Variations in osteoblastic differentiation for hBMSCs selected on Ti surfaces (SMO = smooth; SLA = sandblasted and acid etched; SLV = hydrophilic sandblasted and acid etched) compared to their control, on TCP, were evaluated by genetic expression of bone physiology related genes and functional evaluation of phenotypic characteristics (proliferation, CFU formation, apoptosis effects and quantitative assessment of calcium deposition).

hBMSCs from three unrelated donors (Caucasian, male: 20–30 year age group) and all 3 were used for all the experiments reported here in order to get a good biological spread. The cells were obtained from the Institute for Regenerative Medicine, Texas A & M Health Science Centre, College of Medicine, USA. These cells were isolated from bone marrow aspirates on their ability to adhere to tissue culture plastic compared to non-adherent haematopoietic adult stem cells. The hBMSCs had been pre-characterised for colony forming units, osteogenic, chrondrogenic and adipogenic differentiation and the expression of stromal cell surface markers. The hBMSCs were cultured according to the parameters suggested in previous publications (Dalby et al., 2007, Olivares-Navarrete et al., 2010, Colter et al., 2001). Cells were expanded at low density (100 cells·cm^− 2^) in Growth Medium (GM) comprising Minimal Essential Medium alpha (Gibco) supplemented with 10% lot selected foetal calf serum (Gibco), 1% antibiotics (Penicillin/Streptomycin: PAA laboratories) in a 150 cm^2^ Tissue Culture (TC) treated flask (Nunc) at 37 °C and 5% CO_2_ with bi-weekly medium changes. On obtaining 80% confluence, cells were sub-cultured using a solution of 0.05%/0.002% Trypsin/EDTA in Ca^2 +^/Mg^2 +^ free PBS (PAA laboratories) onto 24 well TC plates containing the following surfaces — TCP, SMO, SMOd, SLA and SLV Ti at a density of 40,000 cells/well. The populations of cells exposed to Ti are the selected populations and contain cells that are phenotypically altered, after selection these cells were expanded using the same method as for the hBMSCs. The population exposed to TCP represent the unchanged parent population, identical in composition to the original MSC population. Phenotypic analysis experiments were performed on selected populations and the parent population cultures that had undergone a maximum of five passages. An experimental matrix is shown in Fig. 1.

Osteogenic differentiation was induced by culturing cells in Osteogenic Medium (OM) comprised of Dulbecco's Modified Medium — Low Glucose (1 g/L) (PAA Labs; UK) supplemented with 10% FBS, 1% antibiotics P/S, 10 nM dexamethasone (Sigma, UK), 10 mM β-glycerolphosphate (Sigma, UK) and 50 μM Ascorbate-2-phosphate (Fluka, UK).

**Fig. 2:**
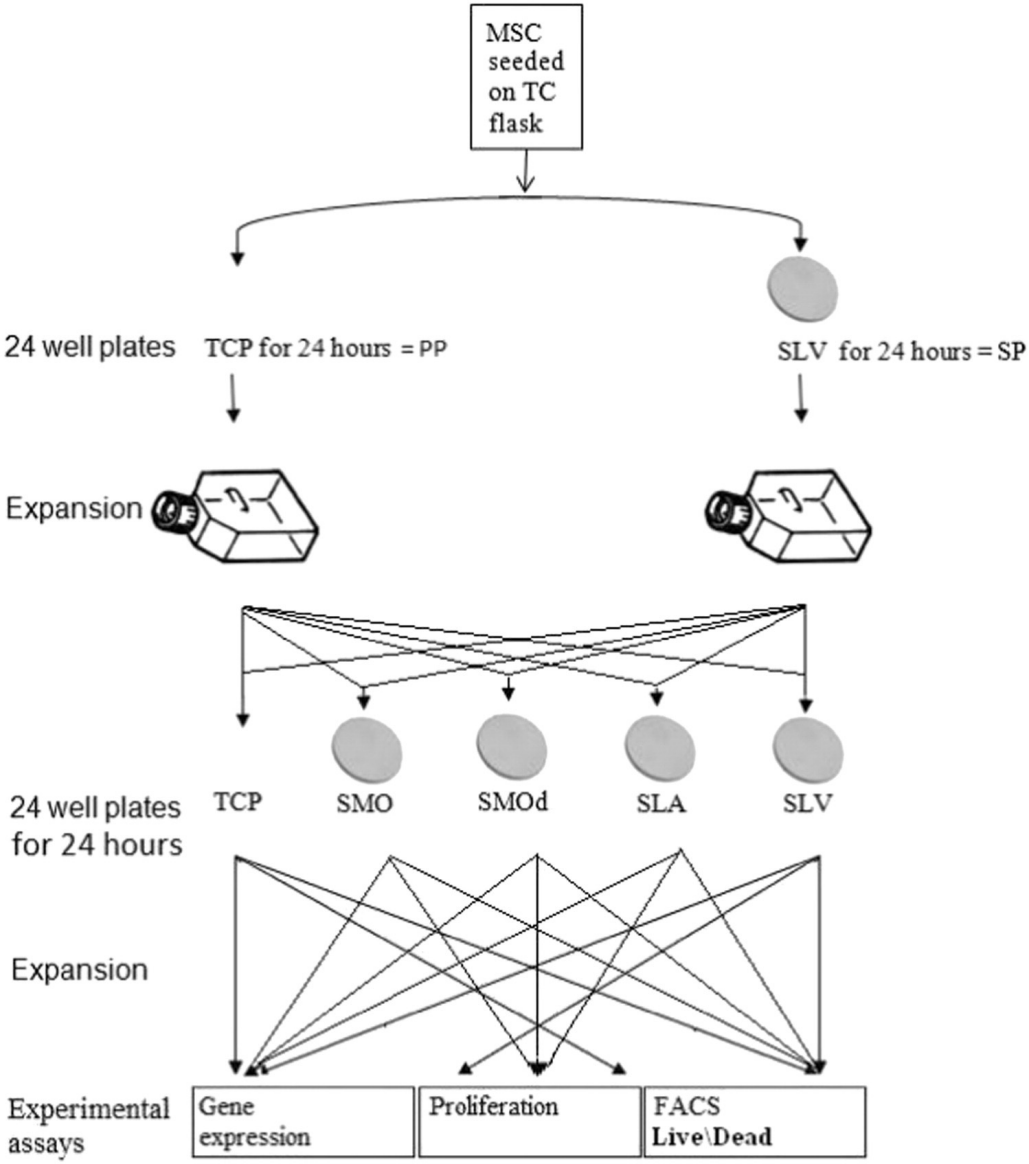
Experimental matrix to determine population changes in the SLV selected population (SP) in comparison to the parent population (PP). Cells that were selected on SLV Ti were ‘parachuted’ back onto Ti surfaces and the variations in osteogenic differentiation were evaluated by genetic expression of bone physiology related genes and functional evaluation of phenotypic characteristics (proliferation, apoptosis effects and quantitative assessment of calcium deposition) using the unaltered parent population as a control.

In order to analyse the population change due to SLV exposure, the cells were re-grown on SLV and the other Ti surfaces using TCP as a control; the selected population's cells were harvested form SLV and then ‘parachuted’ back onto the surfaces for 24 h. Phenotypic analysis experiments were then performed for proliferation, apoptosis and gene expression and compared to the parent population's responses to the same surfaces. This experimental matrix is shown in Fig. 2.

### 2.3 Apoptosis

The levels of apoptosis and necrosis of cells placed on each modified Ti surface and TCP were analysed by staining with Annexin V — FITC and Propidium Iodide (PI) (BD Biosciences). Cells were detached using Trypsin/EDTA that was deactivated by culture medium before being washed twice in PBS by centrifugation. Cells were resuspended in 500 μL 1 × binding solution/sample (10 × binding buffer in 450 μL deionised water). Staining was achieved using 5 μL of Annexin V and 10 μL of PI. Samples were incubated for 10 min in darkness before being analysed by Flow Cytometry (FACScan™ Becton Dickinson). Settings were adjusted to avoid superimposition between FITC and PI.

### 2.4 Proliferation

Proliferation of hBMSCs was determined by seeding cells at a concentration of 1000 cells/well in 1.96 cm^2^ TC-treated 24-well plates containing FBS-typed GM. Samples were evaluated at time points 1, 3, 7 and 10 days post-seeding. 10% *v*/v of AlamarBlue oxidised dye was added per medium and the samples incubated in humidified conditions for a 5 h period. Duplicate 100 μL aliquots of culture supernatant were removed and fluorescence read at excitation 530 nm and emission 590 nm (Floroskan fluorometer). Culture medium was then replaced and cell numbers obtained by extrapolating fluorescent values from a 7 point standard curve (R^2^ = 0.979, highest point of 1.6 × 10^5^ was set up in parallel).

### 2.5 Osteogenic differentiation

Osteogenic-like mineralisation was examined at 7, 14 and 21 days post-seeding by quantifying total matrix calcium (QuantiChrom Calcium) and collagen (Sircol) formation per cell number. Cells from each population were seeded in triplicate at a concentration of 1.25 × 10^4^ cells cm^− 2^ in TC-treated 24-well plates. Cells were stimulated with OM with bi-weekly medium changes. Monolayers were washed 2 × with Ca^2 +^ and Mg^2 +^ free PBS prior to assays. Cell numbers were estimated with AlamarBlue as described above.

Calcium levels were assayed by homogenising a monolayer with 500 μL of 1 M HCl in deionised water for 1 h at room temperature and then assaying a 5 μL aliquot of homogenate with 200 μL of 1 × assay reagent in a clear 96-well TC plate. Absorbance of samples was measured at 405 nm (Tecan). Calcium concentrations (μg/mL) were determined by interpolating absorbance values from a 10 point standard curve. Cells cultured on TCP in GM were used as a negative control of calcium mineralisation.

Extracellular matrix (ECM) collagen was assayed by homogenising a monolayer with 400 μL of cold 0.5 M acetic acid supplemented with 100 μg/mL porcine pepsin (Sigma-Aldrich). Homogenates from three replicate wells were pooled and concentrated overnight with 200 μL of Isolation and Concentration reagent at near 0 °C. The pooling of samples was required due to the minute amounts of collagen formed by cells in individual wells. Concentrated samples were centrifuged to pellet collagen while the albumen rich supernatant was discarded. Collagen pellets were stained with Sirius red in picric acid and later washed in acid-salt solution to remove unbound dye. Bound stain was eluted with 250 μL of an alkali solution. Absorbance of 200 μL of each sample was measured at 555 nm in a clear 96-well microtitre plate. Total quantities were determined by interpolating absorbance values from a 6 point standard curve (R^2^ = 0.998, highest point = 45 μg collagen).

### 2.6 Colony forming units (CFUs)

After expansion, cells from each population were seeded separately at a concentration of 1 × 10^2^ cells per 75 cm^2^ in six T75, TC-treated flasks containing GM. These were cultured, with twice weekly medium changes, for a period of 21 days until cell colonies become apparent. Cultures were fixed and stained in a 3% solution of crystal violet in neat methanol, washed several times with deionised water and air-dried. Images of flasks were taken by camera and analysed using ImagePro software (Alpha Innotech Digital imaging system; UK). Stained colonies were examined and the size distribution determined for each population. Mean diameter was calculated in 2 degree radial increments from the centre of each colony. This gave a better indication of size for irregularly shaped colonies. The number of colonies 2 mm or larger in diameter divided by cells plated × 100 was recorded as the % CFU.

### 2.7 CD surface cell marker analysis

Flow cytometry (FACS) was used to study the relative presence of multiple stem cell surface markers, Stro-1 (FITC), CD146 (V450), CD105 (Brilliant violet), CD164 (PE), CD49d (PE-Cy5), Live/Dead (aqua) within MSC populations. The cytometer was equipped with Violet/blue 405 nm (50 mW) and 488 nm (20 mW) lasers. There are three emission channels per laser which allow for six-colour analysis in addition to forward and side scatter data collection. Cells were detached by Trypsin/EDTA and washed twice in PBS by centrifugation for 5 min at 1300 rpm. Typically, 1 × 10^6^ cells were resuspended in 100 μL of Flow Buffer. The minimum volume of antibody required was deduced by titration and then added to each tube. Volumes used were: Stro-1 (20 μL), CD146 (5 μL), CD105 (5 μL), CD164 (20 μL), CD49d (15 μL) and Live/Dead (1 μL). Samples were incubated for 30 min in the dark then diluted in 1 mL of focusing fluid before being analysed by an Attune Acoustic Focusing Cytometer (AppliedBiosystems).

### 2.8 Gene expression

Transcriptional changes were observed in the apoptosis gene Caspase3 (Casp3), Col1A1 coding for a major component of type 1 collagen, osteogenic regulator Runx2 and extracellular matrix protein osteopontin (OP). hBMSC selected populations from different surfaces were examined after a 24 h seeding and subsequent expansion with real time PCR using the comparative Ct method which compares gene expression levels after normalising to levels of the house keeping/control gene. Cells were seeded at a density of 2.0 × 10^4^ in 24 well plates in CGM. Total RNA was extracted with the Qiagen RNeasy mini kit according to the manufacturer's instructions. RNA preparations were quantified in a Tecan M200 spectrophotometer at λ 260 nm:λ 280 nm absorbance wavelengths. The RNA samples were converted to 100 μL cDNA by using a high capacity cDNA reverse transcription kit (AppliedBiosystems) according to the manufacturers' instructions. Real time PCR system 7300, AppliedBiosystems were performed in 20 μL reactions with 2.0 μL (10 ng of RNA equivalent) of cDNA per reaction. Ct values were normalised to the GAPdH housekeeping gene and calibrated to ΔCt values of cells in suspension (at zero time) to obtain relative fold value of expression.

In order to compare the surface effects of Ti on both the parent population and the selected population from SLV (matrix; Fig. 2), the background expression of genes was measured in the unaltered hBMSCs at time zero (T0). The ΔΔCt values for individual gene expression of the parent population at T0 were given the value 1.00. All relative fold up- or down-regulation was normalised to the parent population T0 = 1.00. This allowed a direct comparison to be made between the background gene expression in the unaltered parent population and in the SLV selected population (T0) as well as the gene expression changes in each population in response to Ti surface effects.

### 2.9 Statistical analysis

Human BMSCs from three donors (N = 3) were used in triplicate (n = 3) throughout the study. Statistical analysis was carried out using Student's t test in GraphPad Prism software (v5.04) with p < 0.05 deemed to be statistically significant.

## 3 Results

### 3.1 Apoptosis

There was a greater effect of necrosis and apoptosis in response to hBMSCs having been exposed to SLV. There were a significantly higher number of dead cells on SLV than on any other Ti surface and TCP.

**Fig. 3:**
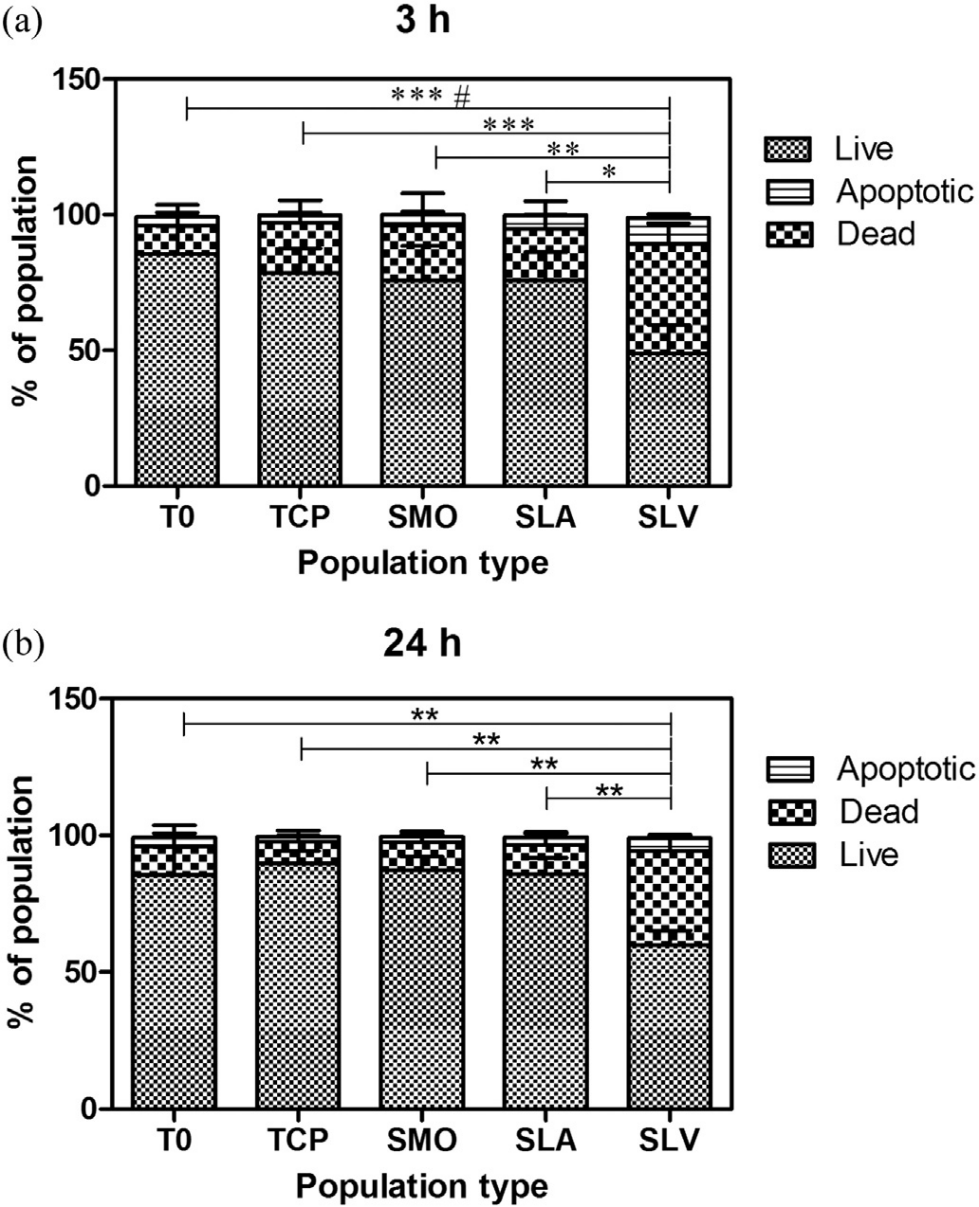
The apoptosis events of hBMSCs at (a) 3 and (b) 24 h on Ti surfaces were measured from FACs analysis using Annexin V/PI Live/Dead assay. The proportion of apoptotic cells is significantly higher in the MSC population from SLV at both time points. Each population showed an increase in live cell numbers from 3 to 24 h. Each bar represents mean ± SD, n = 3. *** = p < 0.001 T0 & TCP vs SLV apoptosis. ** = p < 0.01 SMO vs SLV apoptosis. * = p < 0.05 SLA vs SLV apoptosis. ^#^ = P < 0.05 T0 vs SLV Dead.

The later time point of 24 h showed that there was a higher amount of live cells on all surfaces compared to 3 h (Fig. 3b & a respectively). This suggests that apoptosis on Ti is an initial event followed by a slow recovery of the population through cell proliferation. The smallest recovery effect was seen in the population of cells on SLV.

**Fig. 4:**
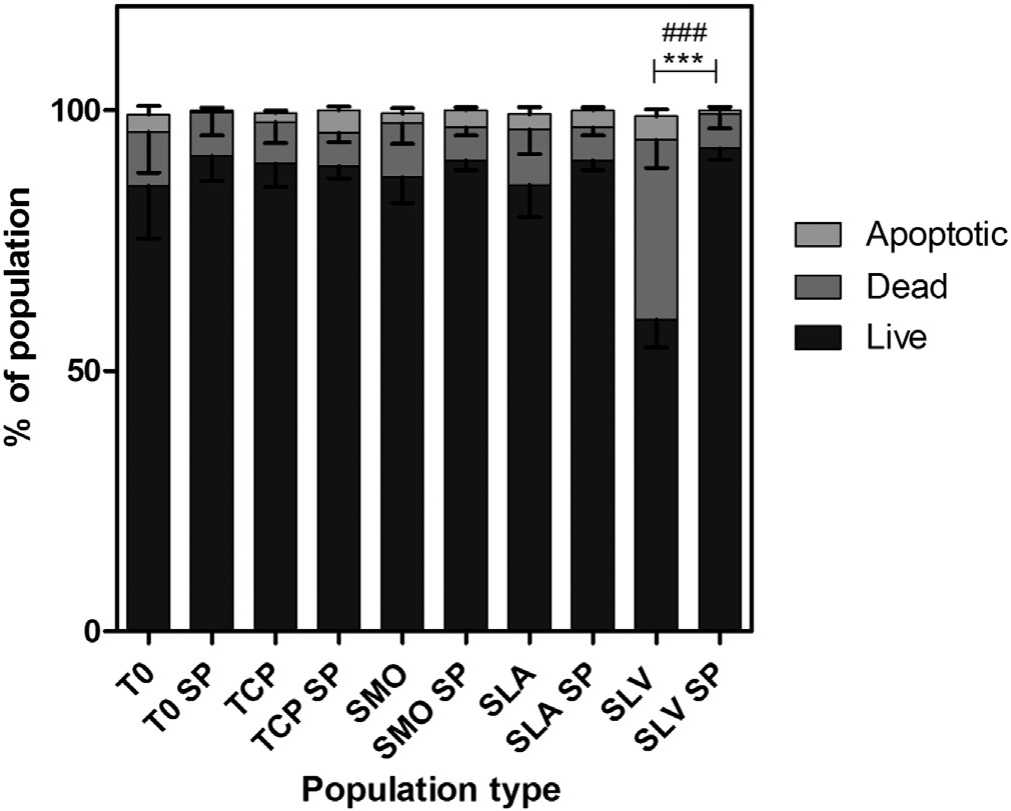
Comparison of apoptosis and necrosis in the selected population (SP) from SLV and the parent population in response to 24 h on each Ti surface. The SLV SP contains more cells that demonstrate the ability to survive the repeated exposure to SLV. Therefore, by taking a cell population selected on SLV and placing it back on this surface, we can suggest that a permanent change in the cell population composition has occurred due to the differential degree of apoptosis that is observed. n = 3, ^###^ = p < 0.001 apoptotic cells and *** = p < 0.001 dead.

The selected population from SLV (SLV SP) showed a significantly higher amount of live cells compared to the parent population from SLV (Fig. 4).

### 3.2 Proliferation

**Fig. 5:**
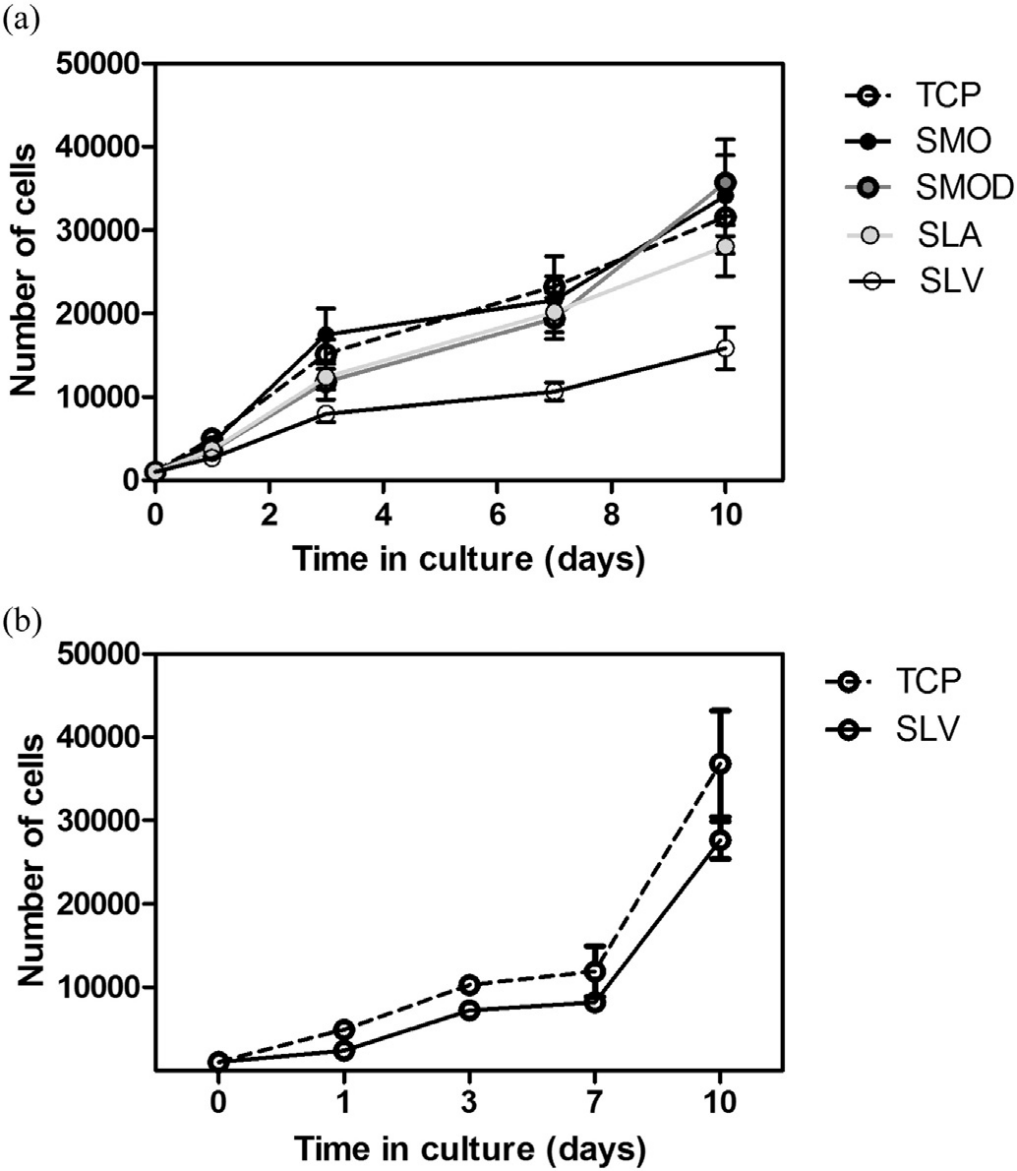
Selected population proliferation was serially assessed with AlamarBlue on TCP. (a) Cells proliferated at a higher rate in the populations from TCP, smooth Ti (SMO and SMOd) and rough hydrophobic Ti (SLA). Cell proliferation was relatively restricted in the SLV selected cell population. (b) MSC proliferation on SLV. The SLV selected population is proliferating at a rate similar to its proliferation on TCP (a). The parent population's proliferation profile (labelled TCP) is reduced compared to that in (a), indicating that a selective process has occurred following 24 h contact with SLV. Each bar represents mean ± SD, N = 3, n = 3.

The cell numbers increased gradually with a greater initial proliferation rate in the parent population (TCP) compared to the selected populations from the smooth and rough Ti surfaces (Fig. 5a). A higher rate of division in the parent population was indicated by roughly a 34-fold increase in cell numbers until a confluent monolayer was reached by day 10. The proliferation on TCP of the selected SLV population was much reduced and showed a cell number increase of roughly half that of the parent population after 10 days in culture.

Analysis of proliferation on SLV shows that the cell population that were selected on SLV and the parent population from TCP have similar profiles (Fig. 5b). The proliferation profile of the parent population on SLV has been reduced from that of the parent population on TCP (Fig. 5a), whereas the selected population proliferation on SLV remains the same as that seen for the selected population proliferation profile on TCP.

### 3.3 Osteogenic differentiation

**Fig. 6:**
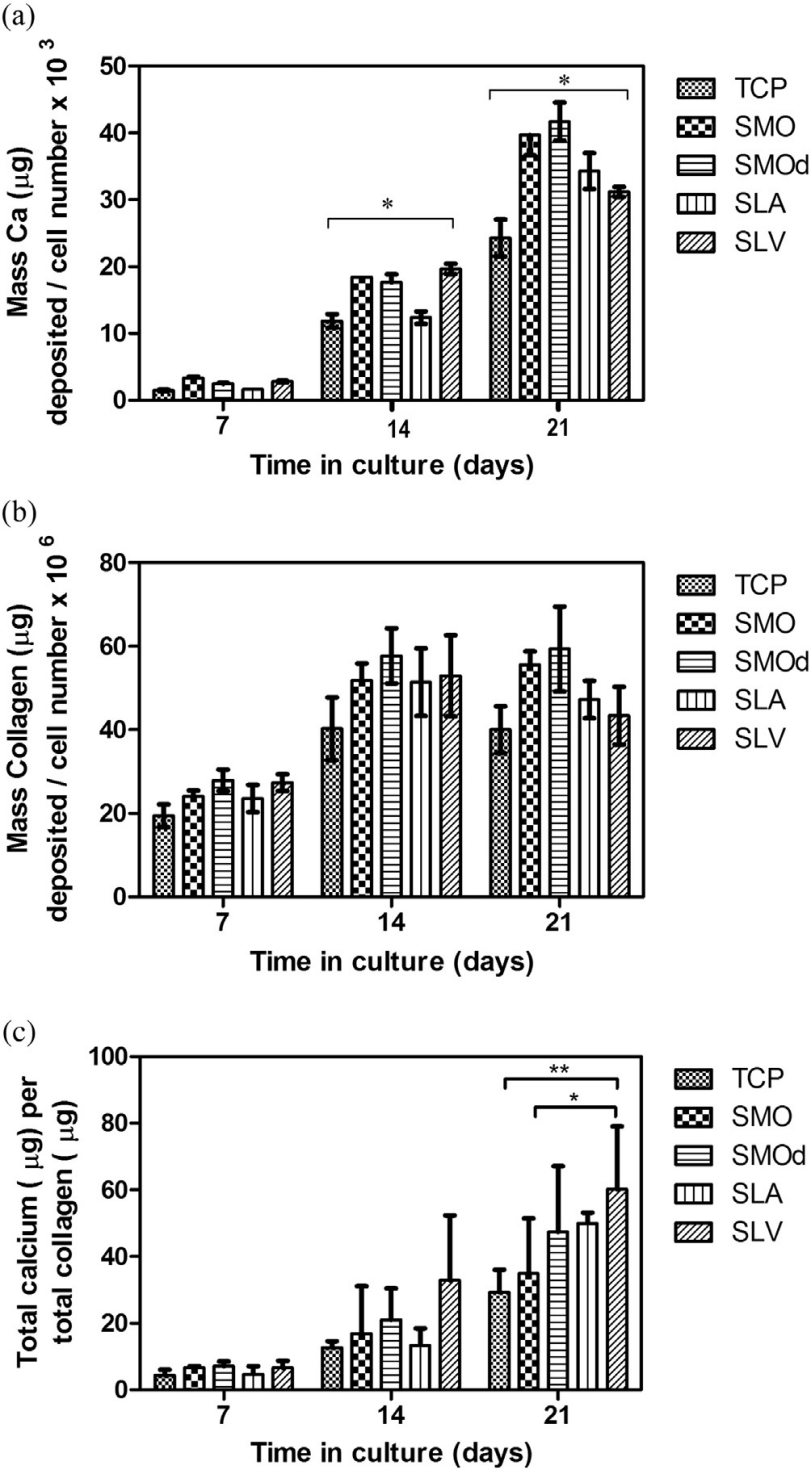
The osteogenic mineralisation in OM of hBMSC populations selected by 24-h exposure to Ti surfaces compared to the unaltered parent population from TCP. (a) Ca^2 +^ mineralisation per cell measured using Quantichrom Calcium assay. * = p < 0.05, TCP 14 vs SLV 14 and TCP 21vs SLV 21. (b) Collagen deposition per cell measured using Sircol Red. (c) Calcium to collagen extra cellular matrix (ECM) ratio formed from each selected population after 24-h selection on modified Ti surfaces. * = p < 0.05 SMO vs SLV 21, ** = p < 0.01 and TCP vs SLV 21. Each bar represents mean ± SD, N = 3, n = 3.

The ECM calcium mineralisation per cell was highest in the SLV population by day fourteen. The amount of calcium deposited per cell is significantly higher on days 14 and 21 than in the parent population of cells (Fig. 6a).

The amount of ECM collagen formed per cell is lowest in the SLV population after day 21 compared to the other Ti surfaces (Fig. 6b). The SMO & SMOd results at day 21 show the cells on the smooth surfaces depositing the most collagen. There is a slight peak in collagen deposition from the rough surface populations at day 14, which then decreases.

In a comparison between the relative ECM compositions deposited per cell for each selected population, significantly higher calcium to collagen ratio was seen in the population selected on SLV at day 21 compared to SMO selected populations and the parent population from TCP (Fig. 6c).

### 3.4 Colony forming units

**Fig. 7:**
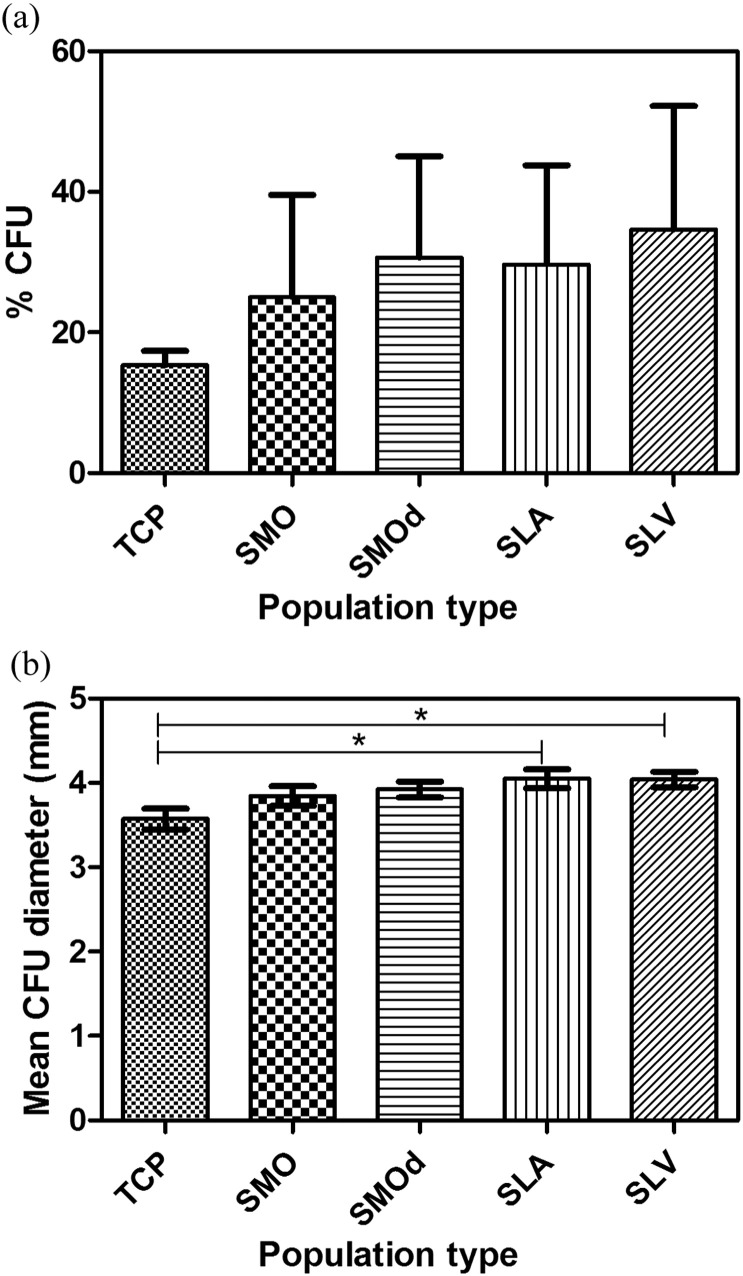
(a) The percentage of CFUs for each selected population in comparison to the parent population on TCP. There is a higher % of CFUs (ns) in the selected populations in comparison to the parent population from TCP. (b) CFU mean diameter distribution in the parent population and the selected populations from modified Ti. Each bar represents mean ± SD, N = 3, n = 3. * = p < 0.05, TCP vs SLA and TCP vs SLV.

Colonies formed in larger numbers and were generally larger for the selected populations from SLV compared to the parent population (Fig. 7a & b). Of 100 cells seeded per flask, over nine replicates, 19% of the parent population cells formed colony units compared to 35% of the SLV selected population. Colonies in the parent population had a mean surface area of 10.95 mm^2^ with an average mean diameter of 3.57 mm. In the SLV population the mean surface area was 12.55 mm^2^ with an average mean diameter of 4.04 mm (p ≤ 0.05 TCP vs SLV).

### 3.5 CD cell surface marker analysis

**Fig. 8:**
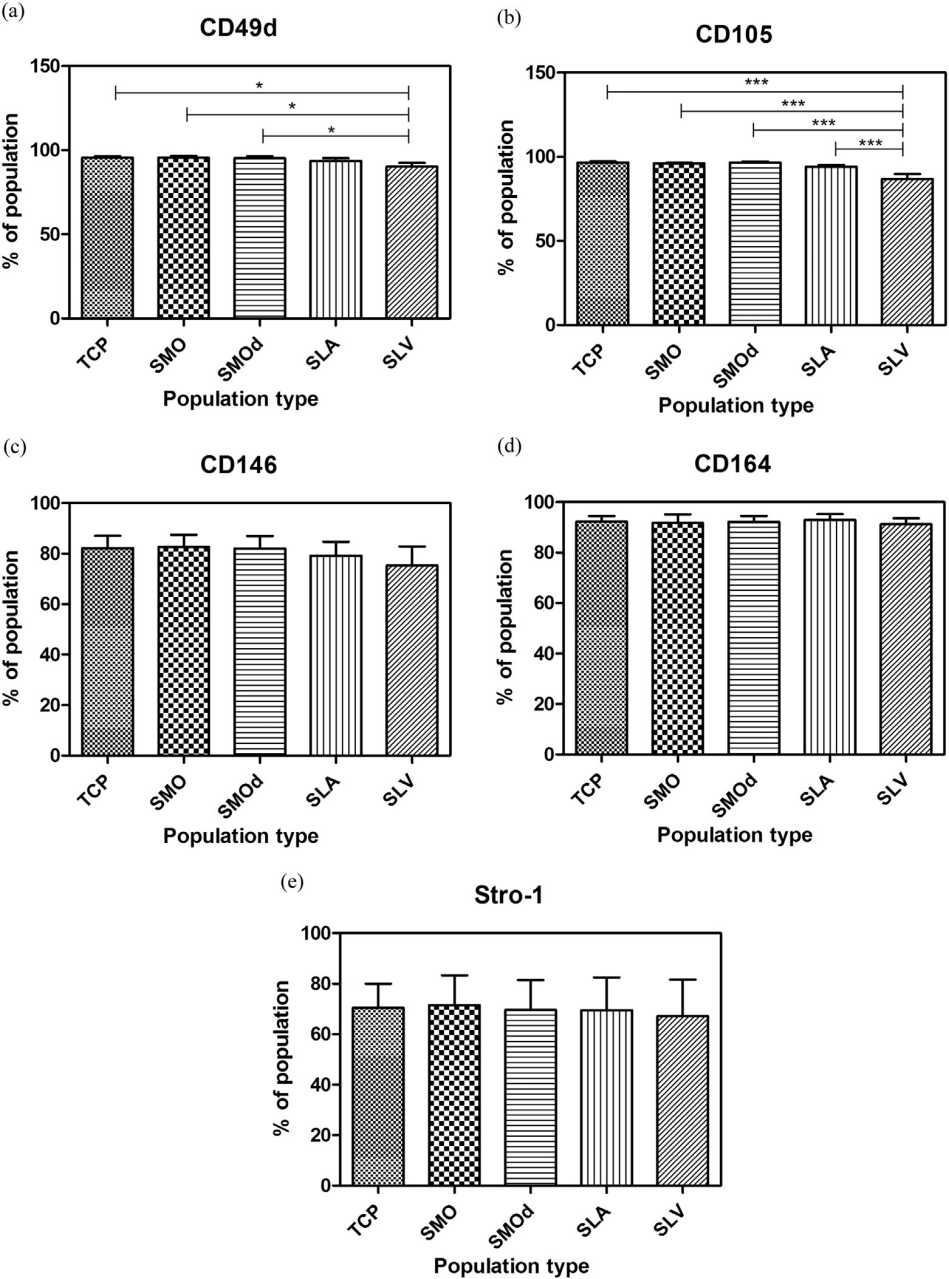
Changes to CD surface cell markers within populations that have been placed on modified Ti for 24 h; (a) CD49d, (b) CD105, (c) CD146, (d) CD164 and (e) Stro-1. n = 3, ***p < 0.001 SLV vs SLA, SMOd, SMO and TCP CD105, *p < 0.05 SLV vs SMOd, SMO, and TCP CD49d.

There was a general trend for cell populations selected on Ti surfaces to show a reduced number of cells with specific stem cell surface CD markers compared to TCP (Fig. 8a-e). SLV selected populations contained significantly lower numbers of cells with CD105 and CD49d surface antigens compared to the parent population and the selected populations from other Ti surfaces.

### 3.6 Osteogenic gene expression

**Fig. 9:**
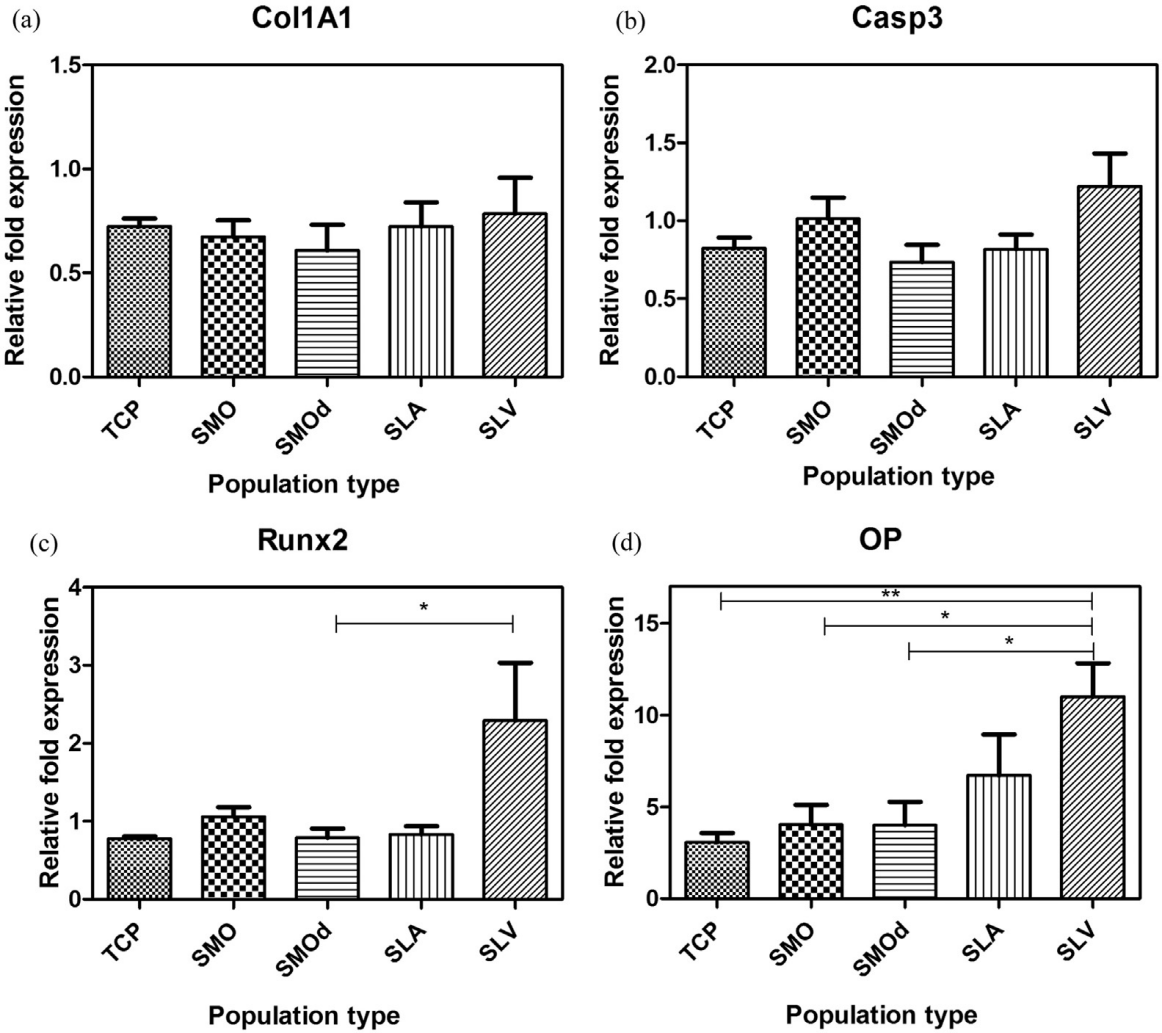
Relative fold changes in the expression of: (a) Col1A1, (b) Casp3, (c) Runx2, and (d) OP in hBMSCs cultured on different surfaces for 24 h in GM. (c) Runx2 was significantly up-regulated on rough hydrophilic surfaces (SLV) compared to smooth hydrophilic (SMOd). (d) OP was significantly expressed at a higher fold by cells on SLV than smooth Ti surfaces and TCP. Each bar represents mean ± SD, N = 3, n = 3. * = p < 0.05, SMO & SMOd vs SLV. ** = p < 0.01, TCP vs SLV.

Up-regulation of target genes was measured in relation to the housekeeping gene GAPdH. Fold values which represent relative gene expression are presented as 2^− ΔΔCdt^ from the Ct method of calculation. Relative gene expression in the populations selected from Ti surfaces was compared to the parent population cultured on TCP and is shown for individual genes in Fig. 9a–d.

The level of Col1A1 expression was not significantly different between the surfaces (Fig. 9a). Caspase 3 was transcribed at a higher level on SLV after 24-h exposure to the surface (Fig. 9b).

The osteogenic transcription factor Runx2 was significantly up regulated in MSCs that had been selected on SLV titanium compared to the population that were selected on TCP (the parent population), SMO and SMOd (Fig. 9c). OP was also up-regulated to a significantly higher extent in the populations from both roughened Ti surfaces (SLA and SLV) compared to the parent population on TCP and the selected populations from smooth Ti (SMO, SMOd) (Fig. 9d).

**Fig. 10:**
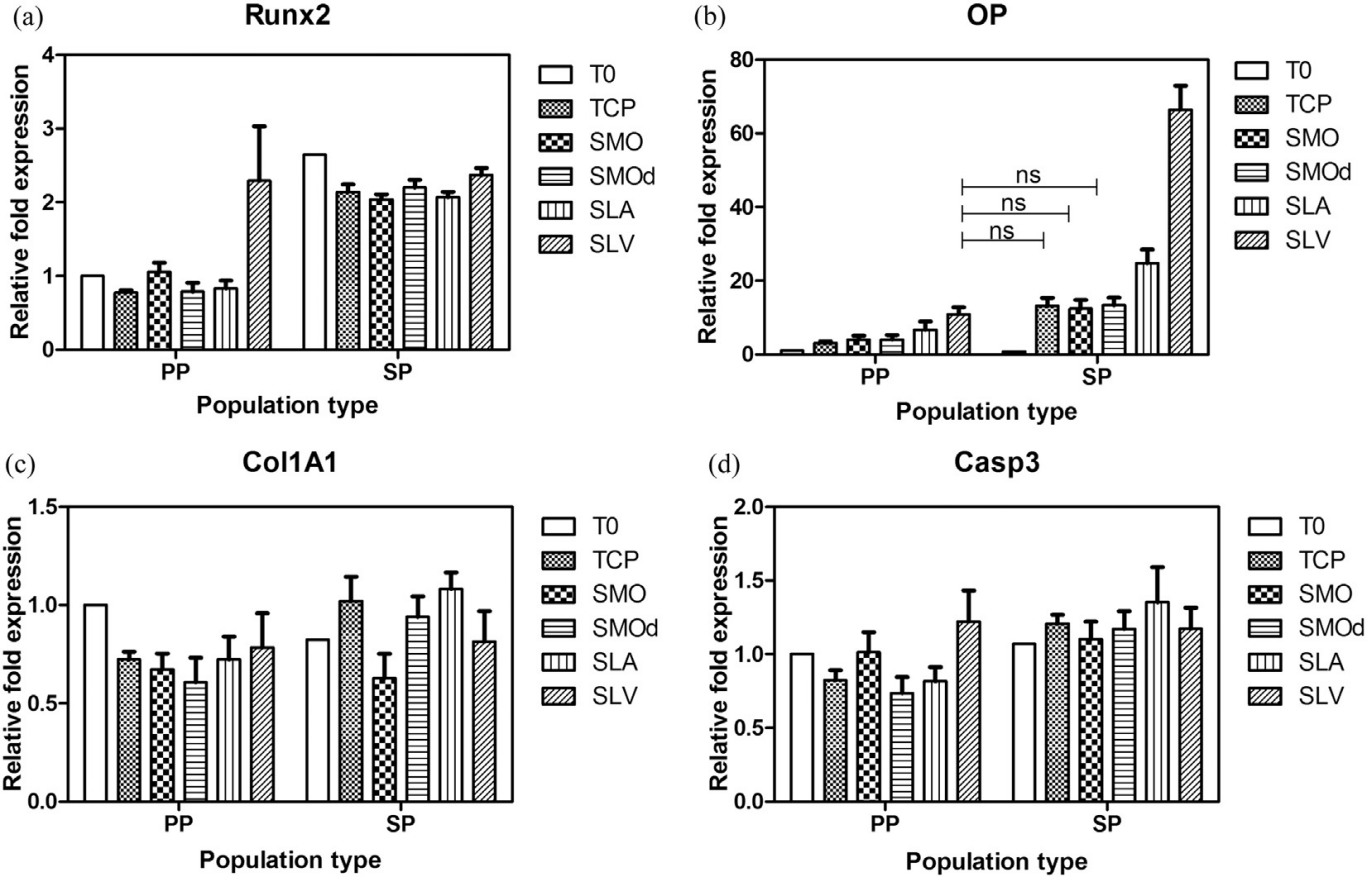
A comparison of gene expression changes, in response to Ti surfaces, between the SLV selected population and the parent population. (a) Cells from the SLV selected population have a 2.64 times higher expression value of Runx2 at T0 compared to the non-selected cells. Within the selected population, there is no significant Runx2 effect in response to subsequent exposure to Ti surfaces. (b) A higher OP response to Ti surfaces is seen in the SLV selected population, in particular, to SLA and SLV. This population responds to TCP, SMO and SMOd Ti in a way similar to that of the parent population genetic response to SLV. (c) Col1A1 and (d) Casp3 expression showed no significant difference between the selected and parent populations on each Ti surface. Each bar represents mean ± SD, N = 3, n = 3. Expression values are normalised to the parent population T0 = 1.00.

Cells from the SLV selected population had a 2.64 times higher expression value of Runx2 at T0 compared to the non-selected cells (PP T0). This suggests that a greater osteogenic commitment potential exists within the SLV selected population. Within this selected population however, there was no significant Runx2 effect in response to subsequent exposure to Ti surfaces (Fig. 10a).

A significantly higher OP response to Ti surfaces was seen in the SLV selected population, in particular, when selected cells were cultured back onto SLA and SLV. It is interesting to note that this population responds to TCP, SMO and SMOd surfaces in a way similar to the parent population's OP response to SLV (labelled ns, Fig. 10b).

No significant differences in the expression of Col1A1 and Casp3 were seen between the Ti selected and parent populations (Fig. 10c and d respectively).

## 4 Discussion

The properties that characterise stem cells are the ability to self-renew and multipotency giving rise to at least three different lineages including osteoblasts, adipocytes and chrondrocytes (Caplan, 2008). In this study, we have used specific assays to investigate the stem-like nature of the cells within selected populations from modified Ti surfaces based on these fundamental characteristics (Cuomo et al., 2009, Davies, 1998, Davies, 2003, Li et al., 2008).

It has been previously observed, in studies by Wall et al. and Olivares-Navarrete et al., that MSCs on Ti substrates have reduced cell numbers compared to TCP (Wall et al., 2009, Olivares-Navarrete et al., 2010). The first response, following contact with Ti, is an increased occurrence of apoptosis and necrosis within the cell population. This is a particularly significant event on SLV compared to TCP and smooth Ti surfaces (p ≤ 0.001). The number of dead cells on SLV at 3 h was significantly higher than at 24 h, indicating that the early apoptotic event was more severe. In addition, the recovery of proliferative capacity was correspondingly slower in the SLV population than the other surfaces studied. Apoptosis appears to be an initial response that does not occur throughout the whole time period that cells spend on the surface. The amount of live cells increases from 3 h to 24 h in all populations. There was a decrease in the significant difference in apoptotic cells across selected populations from 3 h to 24 h (p ≤ 0.001 to p ≤ 0.01 TCP, SMO vs SLV). The dead cells detach from the surface, and the surviving cells proliferate.

Proliferation at each cell cycle is coupled to cell growth. This is because mitotic rate depends on growth (Pardee, 1989). Analysis on TCP, for cells plated onto modified Ti for 24 h prior to the growth experiment, showed that the largest drop in proliferative capacity is seen in the selected population that was exposed to SLV. The proliferation rate was reduced by roughly one half in comparison to the parent population over a ten day period. The decrease in cell growth of the SLV population from the first day in culture on TCP suggests that the more proliferative cells, within the heterogeneous parent population, have been selected out through apoptosis and necrosis following exposure to SLV titanium. This indicates that a selective process has occurred following 24-h contact with SLV.

When both populations are placed on SLV a similar proliferation profile is seen in the selected and parent population (Fig. 5b). There is a selective pressure exerted by the SLV surface acting on the parent population, which reduces its proliferation rate. However, the early proliferation in the selected population is the same as previously seen on TCP. There is no further selective pressure occurring to reduce the proliferation of this population.

There were increased levels of Ca^2 +^ mineralisation and collagen deposition per cell seen in the SLV selected population compared to the parent population when cultured in OM (Fig. 6a & b). This results in significantly higher calcium to collagen ratio deposited per cell (Fig.6c), indicating that a more mineralised ECM structure is being formed. Although it has often proven difficult to draw conclusions about in vivo effects from in vitro studies (Wennerberg & Albrektsson, 2009), this observation could suggest increased bone formation resulting in faster repair processes and potentially increased bone strength. The cells in this population are more likely to differentiate into osteoblasts that are able to deposit a greater amount of mineral, increasing bone strength and reducing the amount of collagen that can result in unwanted movement and possible activated immune response surrounding an implant.

The osteogenic differentiation experiments show that there was a significantly increased concentration of cells within the SLV population that are able to mineralise in OM. Exposure of MSCs to modified Ti surfaces selects cells that are able to differentiate along the osteogenic cell line. The parent population contains a mixture of cell types some of which are more immature ‘stem cell’ like populations and some partially differentiated along one of the multiple lineages that these cells have the potential for. From this data it is uncertain which of these cells are more likely to undergo apoptosis on exposure to Ti surfaces but the CFU work suggests that the ‘stem cell’ like and osteogenic cells survive. Fukiage et al. found that within a group of 100 MSC clones only five of them showed tri-directional differentiation, 78 clones showed the potential to differentiate into either one or two lineages and 17 clones did not show any differentiation potential (Fukiage et al., 2008). This study has shown that contact with SLV Ti increases apoptosis, possibly among the clones that cannot differentiate along the osteogenic route. The resultant progeny of stem cell division may have lost the capacity for self-renewal, but differentiate preferentially yielding osteoblast like cells i.e. more osteogenic cells survive leading to an enriched population. This process is likely to enter into a series of terminal divisions finally yielding an organised tissue such as that in bone growth or repair. This presents the possibility of clinical applications for bone regeneration.

The ability of an adherent population to form colonies of spindle-shaped cells is a phenotypic characteristic attributed to MSCs (Kolf et al., 2007). As such, this property can be used for studying the stem-cell like nature of hBMSCs in culture. The size distribution in the SLV population was skewed towards larger colonies. Colony size is likely to be a function of both cell size and cell spreading. Considering that all the populations' CFUs were cultured on TC-treated flasks, the extent of cell spreading should remain identical. The observed difference in colony size indicates that the individual SLV selected cells, within the colony, tend to be larger than those in the parent population.

We would expect a population enriched in stem cells to display an increased ability of forming colonies and this was seen from the higher % of CFUs produced from the populations selected on SLV. It appears that the number of ‘stem’ like cells has been increased by contact with the Ti surface.

The hBMSCs were tested for a range of cell surface markers (performed by the Institute for Regenerative Medicine, Texas A & M Health Science Centre, College of Medicine, USA). The cells were positive for markers known to be expressed on MSCs i.e. > 90% of cells expressed CD29, CD49c, CD59, CD73a, CD90, CD105, CD147, CD166 and HLA-1. However, a distinct MSC-defining surface marker is far from being established (Pilz et al., 2011).

CD49d is known to have a role in cell–cell interactions as well as cell adhesion to the extracellular matrix (Kamata et al., 1995). CD105 (endoglin) is a regulatory component of TGF-β receptor complex. It is known to be expressed on MSCs and has been used to isolate osteogenic cells (Aslan et al., 2006, Abdallah and Kassem, 2008). On a cellular level, CD105 mediates the response to TGF-β 1 and it has been proposed that endoglin is involved in the cytoskeletal organisation affecting cell morphology and migration (Sanz-Rodriguez et al., 2004). CD146 was found by Pilz et al. to be expressed at a low level in placenta-derived MSCs (pMSCs) and that their corresponding osteogenic differentiation potential was weak. By comparison bone marrow derived MSCs expressed CD146 at high levels and showed pronounced osteogenic differentiation potential (Pilz et al., 2011). CD164 is known to function as a cell adhesion molecule. CD164 positive cells have been shown by Braun et al. to have a strong potential to differentiate into osteoblasts as characterised by von Kossa and Alizarin Red-S staining (Braun et al., 2010).

Our findings from CD surface markers, although in slight contrast to the CFU results, do not represent a major change in cell population composition. The small, significant reduction in the amount of CD105 and CD49d positive cells in the SLV population suggests that the cells may be different in character but this is not an indication of stem cell or osteogenically relevant changes. This is particularly the case for Stro-1, whose surface expression levels were not significantly altered between the populations, whereas a study by Li et al. found that osteoprogenitor cells have been shown to have a positive link between surface expression of Stro-1 (Gronthos et al., 1994).

The process of adaption begins with the genes. A new surface environment, such as titanium, provides a stimulus that turns specific genes on or off. By altering the expression of genes, the surface environment changes the rates on which the cell makes or breaks down specific proteins. For example, roughened surfaces turn on genes for the production of Caspase 3 proteins. An increase in the transcription of Caspase 3 proteins within the cells has been known to lead towards programmed cell death (Porter and Janicke, 1999, Liu et al., 1997). The cells and their transcription systems adapt over days and weeks to the cumulative effects of a new environment.

There was an up-regulation in the cell population from SLV for the transcription factor Runx2 and for Casp3, which up-regulate several downstream entities and that play a role in osteogenic differentiation and apoptosis respectively (Ziros et al., 2008). The gene for OP, whose transcription protein is involved in calcium mineralisation through the mediation and assembly of calcium nucleation sites (Franz-Odendaal et al., 2006), was also significantly up-regulated in the SLV selected population.

The up-regulation of Runx2 and OP provides an indication of a commitment towards the osteogenic lineage in populations following exposure to roughened, hydrophilic titanium.

Col1A1 is a widely expressed gene. It is involved in the formation of type 1 collagen and is known to form a template for the mineralisation of bone (Pace et al., 2001). However, its expression was not changed much in any of the selected populations compared to the parent population. The up-regulation of Caspase 3 indicates that higher apoptosis occurred within cells of the SLV compared to the parent population.

The gene expression data here confirms our results from the mineralisation and proliferation experiments. It suggests that there are increased levels of cell death in response to SLV and increased differentiation along the osteogenic pathway. It appears that the roughness and hydrophilicity of the surface has an impact on both reducing the cell number and also directing the remaining MSCs towards the osteogenic lineage.

The higher gene expression of Runx2 at T0 within the selected population, indicates that either more cells are expressing this gene or that the same number of cells are expressing but at a higher level (Fig. 10a). It is possible that a more concentrated population of osteogenic cells has been formed from exposure to SLV, following cell death of those that are non-osteogenic. This selected population has no further Runx2 response to modified Ti surfaces, which indicates that the gene cannot be up-regulated further. This provides additional evidence that the population change is permanent. It is possible that the differentiation event has occurred and cannot be repeated by further exposure to SLV. These cells, however, are pre-programmed to respond osteogenically and they produce much higher levels of OP in response to a surface, such as that on TCP. OP expression is then further increased in response to Ti surfaces, in particular SLA and SLV, which are known to trigger associated mineralisation through increased calcium nucleation sites (Franz-Odendaal et al., 2006) (Fig. 10b).

It is likely that a sub-population of stem cells which differentiate to form osteogenic cells on SLV has been enriched. This is supported both by the increased amount of osteogenic differentiation seen in the SLV selected population after 14 days in OM and the increase in apoptosis. The stem cells with osteogenic potential survive on rough hydrophilic Ti and the remainder of the stem cells is removed from the population. This then reduces the density of proliferative stem like cells in the population.

SLV is a surface that induces a high amount of cell death and is also a surface that has shown superior osseointegration in bone implants in vivo (Rupp et al., 2006). Cell death may play a role in osseointegration by selecting the cells from a population that have the potential to differentiate into osteogenic cell lines.

SMO and SMOd populations showed no significant differences between them, in gene expression or calcium mineralisation, indicating that hydrophilicity of the surface alone is not responsible for the enrichment effect. SLV surfaces have the biggest effect on the cell population indicating that a combination of both roughness and hydrophilicity is required.

## 5 Conclusion

Enrichment of osteoblast-like cells from a heterogeneous population of hBMSCs can be achieved through adhesion to roughened, hydrophilic titanium and subsequent expansion. The selection effect can be seen to be largely due to the chemistry of the surface since SLA and SLV have identical surface roughness. It was seen that a population expanded following 24 h adhesion to SLV is enriched in cells that are capable of osteogenic differentiation with some loss in proliferative capacity. On contact with the surface hBMSC numbers are reduced due to increased levels of necrosis and apoptosis, which results in the removal many non-osteogenic cells. This process leads to the formation of a population of cells that possess a natural tendency to differentiate along the osteogenic lineage. Greater ECM calcium to collagen ratios are formed from hBMSCs selected on the SLV with these cells likely to form better quality bone leading to better implant anchoring in vivo and more effective cell selection in vitro for the use in regenerative applications. The data from this study suggests that osteogenic enrichment of hBMSC populations by modified Ti for repair and regeneration is feasible and if successfully translated this would allow the selection and expansion of viable osteogenic stem cells with bioreactors, to provide sufficient biomass to make tailored, homologous replacement bone implants that would result in minimal risk of rejection.

## Ethics

The cells used in this study were ethically obtained from fully consented donors and supplied to us by the Institute for Regenerative Medicine, Texas A & M Health Science Centre, College of Medicine, USA.
